# The acute healthcare burden of periprosthetic fractures in a regional orthopaedic trauma unit

**DOI:** 10.1007/s11845-026-04445-5

**Published:** 2026-05-22

**Authors:** Brian Rigney, Supyae Yadanar, Malak Alwaheed, Aaron Glynn

**Affiliations:** https://ror.org/029sr1j73 grid.417310.0 0000 0004 0617 7384 A. Trauma and Orthopaedics Department, Our Lady of Lourdes Hospital Drogheda, Drogheda, Ireland

**Keywords:** Hip fracture, Periprosthetic fracture, Healthcare resources

## Abstract

**Background:**

Periprosthetic fractures (PPF) are increasing in incidence alongside an ageing population and expanding arthroplasty services. Although PPF are included within national hip fracture databases and share similar standards of care, their greater surgical complexity and resource utilisation is not formally recognised by current frameworks.

**Aim:**

To compare acute inpatient resource utilisation between patients sustaining periprosthetic femoral fractures and those sustaining hip fractures in a regional orthopaedic trauma unit.

**Methods:**

A single-centre retrospective study was performed including 45 consecutive operatively managed PPF cases (January 2022–June 2024), matched 2:1 with 90 consecutive hip fracture patients (January–June 2024). Demographics, frailty, American Society of Anesthesiologists (ASA) grade, time to surgery, operative duration, length of stay, critical care utilisation, and inpatient mortality were analysed.

**Results:**

Baseline demographics were homogenous between groups. Patients with PPF waited significantly longer for surgery (mean delay 1.75 days, p<0.0001) and had longer operative times (mean increase 61.5 minutes, p<0.0001). Length of stay was similar between groups (16.75 vs 15.88 days, p=0.67). PPF patients were less frail but more frequently required post-operative critical care admission (15.6% vs 4.4%, p=0.04). Inpatient mortality was higher in the PPF group, though not statistically significant.

**Conclusion:**

Periprosthetic fractures have a substantially greater acute healthcare burden than hip fractures, particularly in terms of operative time, surgical delay, and critical care utilisation, despite similar mortality rates, lengths of stay and lower frailty scores. These findings highlight the need for recognition of PPF complexity and consideration of enhanced resource allocation for centres managing high PPF volumes.

**Supplementary Information:**

The online version contains supplementary material available at https://doi.org/10.1007/s11845-026-04445-5.

## Introduction

Periprosthetic fractures (PPF) are increasing in incidence alongside an aging population with increased access to arthroplasty specialists [1]. They represent a complex injury that requires specialist input and increased resources. The patients are often frail and comorbid, with prolonged hospital stays, similar to hip fracture patients.

The National Hip Fracture Database (NHFD) is an initiative whereby all hip fractures in the UK are required to receive a standard of care designed to improve patient outcomes [2]. In 2020, the NHFD began collecting data regarding all PPF of the femur. Furthermore in 2022, they acknowledged the similar care needs of these two patient groups, whereby PPFs have been given the same Key Performance Indicators (KPI) as hip fractures [3].In Ireland, the Irish Hip Fracture Database (IHFD) performs a similar role [4]. It sets out 7 standards of care for hip fracture patients, and offers best practice tariffs if all standards are achieved [5]. The database was founded in 2012 and it collects data on fractures from the femoral neck to the subtrochanteric region. Since its inception, it has collected data regarding PPFs that fall within this region, and thus those fractures should be subject to the IHFD standards. However, as the entire femur is not included, there is likely a large number of PPFs not being recorded. Nevertheless, despite their inclusion, there is no specific recognition of the substantially increased complexity associated with these injuries.

The aim of this paper was to compare patients sustaining PPF with those sustaining hip fractures and determine any difference in resource utilisation among these two patient populations. Our hypothesis was that patients with PPF would require extra resources during their inpatient stay. If our hypothesis is correct, it would be prudent to consider additional resource allocation to centres performing larger volume PPF work.

## Methods

This was a single institution retrospective study of a consecutive series of PPF treated in our centre between January 2022 and June 2024 that has been matched with a 2:1 ratio to a consecutive series of hip fracture patients between January 2024 and June 2024. Ethical approval was sought for this study but not required by our institution’s ethics board due to the retrospective nature of the study. It was approved as an audit by the institution’s audit and ethics board (registration number: LH2025/04).

The inclusion criteria in the PPF arm being any PPF of the femur during the study time period that underwent operative management. The inclusion criteria in the hip fracture group being fracture meeting the hip fracture definition set out by the Irish Hip Fracture Database (IHFD) [5].

Demographics including age, sex, fracture pattern, ASA grade, frailty scale were recorded. Outcomes measured included time to surgery, operative time, length of stay, inpatient mortality.

Data sources included theatre logbooks, the institutions electronic patient record, patient charts and the national radiology database. Fractures were analysed as per the Unified Classification System [6] independently by two reviewers. Any disagreements were arbitrated by the senior author.

Statistical analyses were performed using GraphPad Prism Software V10 for Mac. We performed a priori power analysis using an effect size of 0.6, alpha of 0.05 and a target power of 0.8. This showed a required sample size of 45 in each group to detect statistical significance.

## Results

A total of 45 PPFs and 90 hip fractures were included in the analysis. The results are outlined in Table 1. The groups were relatively homogenous in their baseline demographics with no significant difference in mean age (77.6 and 78.7 respectively, *p* = 0.49) and no significant difference in sex (Percentage of females: 42% and 55.6%, *p* = 0.15). Fracture patterns are outlined in Supplementary Tables 1 and Supplementary Table 2.

**Table 1:** Demographics and Results Summary

	PPF	Hip Fracture	*P* value
Age (years)	77.6	78.7	0.49
N Female (%)	19 (42%)	45 (55.6%)	0.15
Mean Operative Time (minutes)	138.4	76.9	< 0.0001
Mean Length of Stay (days)	16.75	15.88	0.67
Mean time wating for operation (days)	3.35	1.6	< 0.0001
Inpatient mortality N (%)	4 (8.9%)	4 (4.4%)	0.3
ICU admission	7 (15.6%)	4 (4.4%)	0.04

There was no difference in mean length of stay (16.75 days vs. 15.88 days, *p* = 0.67). The mean amount of time patients waited for their operation was increased in the PPF group by 1.75 days (*p* < 0.0001). The mean operative time was longer by 61.5 min in the PPF group (*p* < 0.0001).

Although inpatient mortality was higher in the PPF group, this did not reach statistical significance (8.9% vs. 4.4%, *p* = 0.3). However significantly more PPFs required a stay in critical care post-operatively (15.6% vs. 4.4%, *p* = 0.04).

## Frailty and ASA

The distribution of the clinical frailty scale scores differed significantly between the two groups, with PPF patients being generally less frail than the hip fracture patients (*p* = 0.009) (Table 2).

**Table 2:** Clinical Frailty Score

	Very fit	Fit	Well	Managing well	Vulnerable	Mildly frail	Moderately frail	Severely frail
PPF	6	3	7	11	6	5	3	4
Hip Fracture	3	0	10	15	14	11	32	5

*P* value = 0.009 (Fisher exact test)

Although a higher proportion of hip fracture patients were classified as ASA 3 and 4, the difference was not statistically significant (*p* = 0.15) (Table 3).

**Table 3:** American Society of Anesthesiologists (ASA) Classifications

	ASA 1	ASA 2	ASA 3	ASA 4
PPF	2	15	20	8
Hip Fracture	0	22	48	20

*P* value = 0.15 (Fisher’s exact test)

## Discussion

Our study shows that PPF use significantly more theatre time and require ICU care more frequently post-operatively whilst having to wait longer for their operation. The significantly longer wait time for surgery (1.75 days), may be a reflection of the complex nature of PPF management. These fractures are often required to be managed by specialist arthroplasty consultants. Additionally, these fractures, particularly when managed with revision arthroplasty, require specialist implants that may not be readily available. As a result, patients often are waiting for a suitable surgeon and suitable equipment, thus delaying their surgery. Similar disparities in wait times have been reported with PPF waiting up to 2.8 days longer than hip fractures for surgery in the literature [7]. Current guidelines advocate for hip fracture surgery to be performed within 36–48 h, as this has been shown to reduce mortality rates. There has been varying reports on the effect of time to surgery on PPF and outcomes, particularly long-term mortality. Murphy et al. reported a slight increase in 1 year mortality in PPF patients who received surgery after 48 h compared with patients waiting over 48 h, however the difference was not statistically significant (4% vs. 9%, *p* = 0 0.1) [8]. However in a larger study by Wulbrand et al. surgery within 24 h was shown to have a significant reduction in 1 year mortality (8.3% vs. 20.5%) [9].

The longer operative time (138.4 vs. 76.9 min) aligns with the complex nature of these injuries. Often the decision regarding whether to perform an open reduction internal fixation or a revision arthroplasty can only be made intra-operatively once the stem stability has been assessed. Therefore, the surgeon and their team must be ready for all possibilities prior to starting the case [10]. In addition to this, the longer case time for PPF, reduces the amount of available theatre time for other trauma cases. If a centre is performing a large volume of PPF cases, they may need additional resources, including increased access to theatre and critical care. There is a large variability in the management choice for PPF between hospitals, highlighting the complex nature of these injuries, and that there may not be a one size fits all approach to their surgical treatment [11].

Inpatient mortality was higher in the PPF group, however this did not reach significance. This trend may still have relevance, and other studies report a similar rate of early mortality to ours (4.1–9.3%) [12, 13]. This mortality rate climbs at 1 year, ranging from 11.2–40%%, dependent on medical comorbidities and age. Patients age over 80 and a Charlson comorbidity index ≥ 3 being significantly associated with increased 1 year mortality rates, as well as preoperative functional status and length of operative time [13, 14]. Indeed functional outcomes are worse following PPF [15], with over 58% of patients not returning to their preoperative walking ability [13]. Alongside a functional decline, lower psychological well-being following PPF has been reported in a number of studies [16, 17]. This highlights the severity of the impact of these injuries on the individual patient.

The significantly higher rates of PPF patients requiring critical care could indicate a larger physiological impact to the patient and increased need for intensive post-operative monitoring. Additionally, the prolonged surgical time could contribute to this finding. In contrary to this, the clinical frailty scores were higher in hip fracture patients and there was no significant difference in ASA. This may indicate that surgical complexity, combined with the prolonged wait time for the operation as well as longer surgical times could be the reason for critical care admission, rather than comorbidities alone.

Despite the hip fracture patients being a frailer cohort, the length of stay was similar between the two patient groups, indicating that a similar level of comprehensive post-operative care and rehabilitation is required for PPF fracture patients. The length of stay in our study for PPF (16.75 days) is similar to other studies with mean length of stay ranging from 15 to 21 days [9, 11, 13].

As stated previously, PPF often require specialist implants. This, alongside increased theatre time, critical care utility and prolonged lengths of stay can increase the healthcare associated costs. In a 2014 study examining the financial burden of PPF, the average cost of a hospital stay following PPF was of a revision arthroplasty for PPF was $24,831, which increased to $34,078 if revision arthroplasty was required and $91,035 if a distal femur replacement was required [18]. There has been a 13% year on year rise in PPF incidence, so one can infer that the economic costs of these injuries as a collective will continue to grow [19].

### Limitations

The limitations of this study include the retrospective nature of the data. Additionally, the lack of power in the difference in mortality rate may be related to the follow-up being confined to inpatient stay, or sample size. However, the focus of this study is on the impact of PPF in the acute hospital setting rather than in the longer term health system.

There has been limited prospective studies comparing these two populations, however they are now linked via the NHFD and IHFD. Future research should utilise the prospective data available within these databases.

## Conclusion

This study shows that PPF, when compared to hip fractures, utilise more resources in terms of operative time and critical care beds, whilst waiting longer for their surgery. They have a high inpatient mortality rate with similar lengths of stay despite being less frail. These are complex injuries requiring specialist arthroplasty surgeon management. Including these patients in the hip fracture database has been a positive step towards improving care. However, as highlighted in this study, their management requires considerable resource allocation. Therefore, centres performing a large volume of PPF cases should be provided with additional resources in the future.

## Electronic Supplementary Material

Below is the link to the electronic supplementary material.

Supplementary Material 1 (DOCX 13.1 KB)

Supplementary Material 2 (DOCX 13.0 KB)
