# Supplementary material for: The acute healthcare burden of periprosthetic fractures in a regional orthopaedic trauma unit
Source: Ir J Med Sci. 2026 May 22;195(5):2505–8. doi: 10.1007/s11845-026-04445-5 (PMC13740073; doi:10.1007/s11845-026-04445-5)
Supplement: Supplementary file 1 [file 11845_2026_4445_MOESM1_ESM.docx]

| Supplementary Table 1 | |
| --- | --- |
| Hip Fracture pattern | |
| Intracapsular - undisplaced | 24 |
| Intracapsular - displaced | 29 |
| Intertrochanteric | 32 |
| Subtrochanteric | 5 |
| Total | 90 |
