# Supplementary material for: The acute healthcare burden of periprosthetic fractures in a regional orthopaedic trauma unit
Source: Ir J Med Sci. 2026 May 22;195(5):2505–8. doi: 10.1007/s11845-026-04445-5 (PMC13740073; doi:10.1007/s11845-026-04445-5)
Supplement: Supplementary file 2 [file 11845_2026_4445_MOESM2_ESM.docx]

| Supplementary Table 2 | |
| --- | --- |
| Fracture Pattern Periprosthetic Fracture | |
| B1 | 20 |
| B2 | 11 |
| B3 | 2 |
| C | 8 |
| D | 4 |
